# Supplementary material for: Healthy lifestyle and cognitive decline in middle-aged and older adults residing in 14 European countries
Source: Nat Commun. 2024 Jun 27;15:5003. doi: 10.1038/s41467-024-49262-5 (PMC11211489; doi:10.1038/s41467-024-49262-5)
Supplement: Supplementary file 1 — Supplementary Information [file 41467_2024_49262_MOESM1_ESM.pdf]

Healthy lifestyle and cognitive decline in middle-aged and older adults residing in 14 European countries

Supplemental materials

Figure S1. Flowchart of sample selection in ELSA. 2

Figure S2. Flowchart of sample selection in SHARE. 3

Table S1. Characteristics of the pooled analytic sample at study baseline in men (N=14,317). 4

Table S2. Characteristics of the pooled analytic sample at study baseline in women (N=17,716). 5

Table S3. Participants remaining in study at each wave. 6

Table S4. Mean baseline standardised memory performance in each lifestyle. 7

Table S5. Mean baseline standardised fluency performance in each lifestyle. 8

Table S6. Independent associations between behaviours and cognitive decline. 9

Table S7. Difference in memory decline over 10 years between the healthiest lifestyle and other lifestyles after excluding participants who abstain from alcohol consumption (N=22,577). 10

Table S8. Difference in fluency decline over 10 years between the healthiest lifestyle and other lifestyles after excluding participants who abstain from alcohol consumption (N=22,577). 11

Table S9. Difference in memory decline over 10 years between the healthiest lifestyle and other lifestyles after adjustment for practice effect (N=32,033). 12

Table S10. Difference in fluency decline over 10 years between the healthiest lifestyle and other lifestyles after adjustment for practice effect (N=32,033). 13

Table S11. Difference in memory decline over 10 years between the healthiest lifestyle and other lifestyles after adjustment for body mass index (N=32,010). 14

Table S12. Difference in fluency decline over 10 years between the healthiest lifestyle and other lifestyles after adjustment for body mass index (N=32,010). 15

Table S13. Difference in memory decline over 10 years between the healthiest lifestyle and other lifestyles after adjustment for self-reported hearing difficulty (N=32,033). 16

Table S14. Difference in fluency decline over 10 years between the healthiest lifestyle and other lifestyles after adjustment for self-reported hearing difficulty (N=32,033). 17

Table S15. Countries included in ELSA and SHARE. 18

Figure S1. Flowchart of sample selection in ELSA.

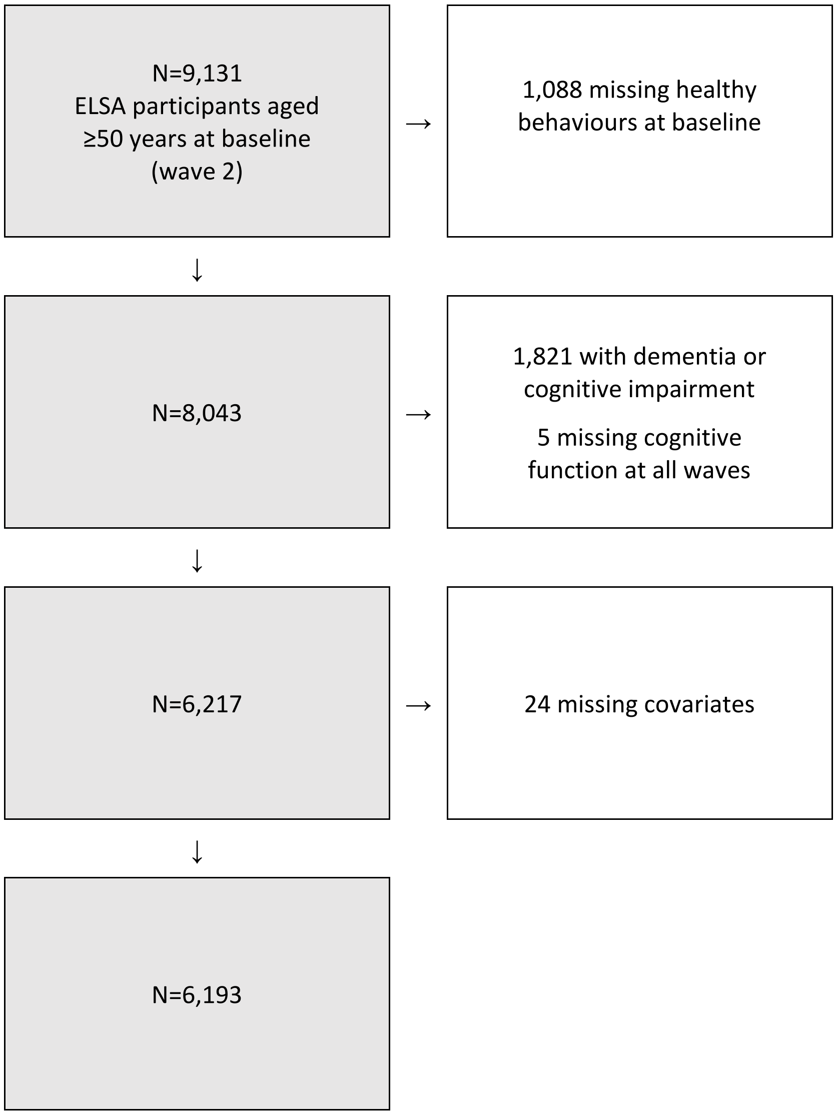

Figure S2. Flowchart of sample selection in SHARE.

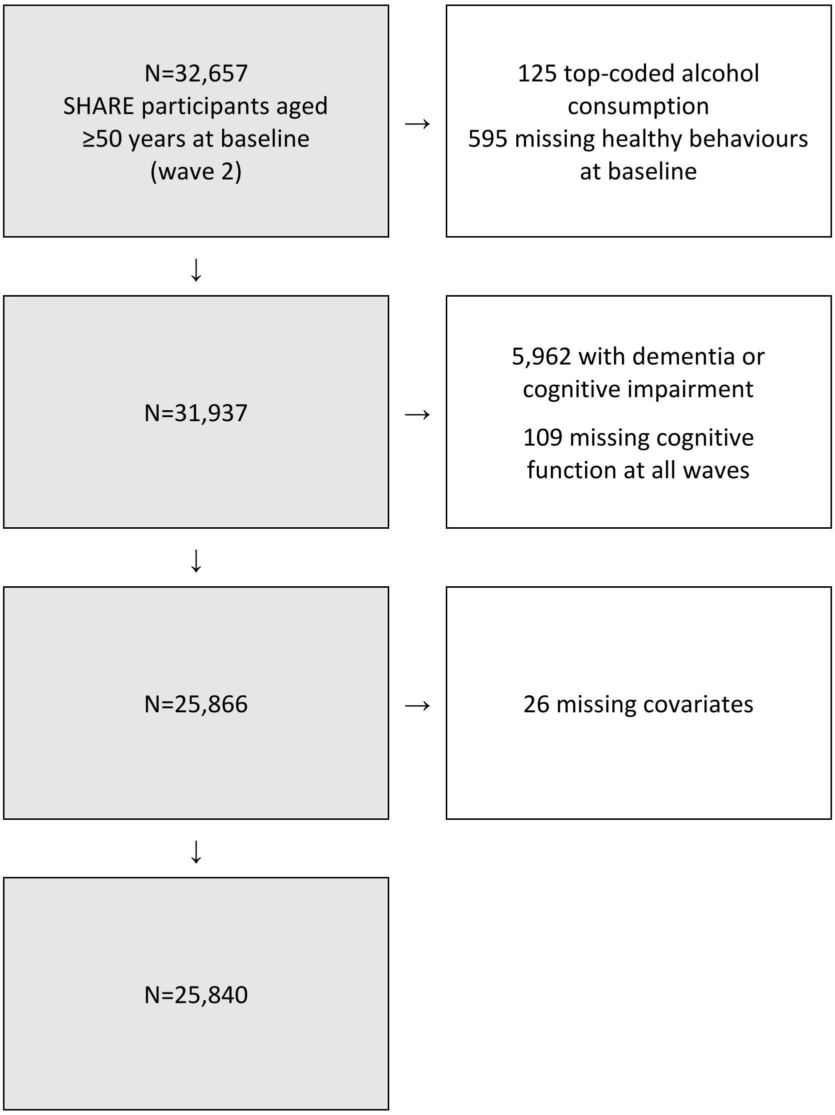

Table S1. Characteristics of the pooled analytic sample at study baseline in men (N=14,317).

	**Non-smoking**			**No-to-moderate alcohol consumption**			**Weekly MVPA**			**Weekly social contact**		
	No N = 3099	*Yes* N = 11218	P-value	*No* N = 4743	*Yes* N = 9574	P-value	*No* N = 7416	*Yes* N = 6901	P-value	*No* N = 7915	*Yes* N = 6402	P-value
Age at baseline, mean (SD)	61.1 (8.2)	65.6 (9.6)	<0.001	62.4 (8.5)	65.7 (9.8)	<0.001	66.7 (10.0)	62.4 (8.4)	<0.001	65.1 (9.9)	64.0 (9.0)	<0.001
Education												
Below upper secondary	1288 (41.6)	4345 (38.7)		1621 (34.2)	4012 (41.9)		3230 (43.6)	2403 (34.8)		3626 (45.8)	2007 (31.3)	
Upper secondary	1222 (39.4)	4230 (37.7)	<0.001	1925 (40.6)	3527 (36.8)	<0.001	2701 (36.4)	2751 (39.9)	<0.001	2821 (35.6)	2631 (41.1)	<0.001
Tertiary	589 (19.0)	2643 (23.6)		1197 (25.2)	2035 (21.3)		1485 (20.0)	1747 (25.3)		1468 (18.5)	1764 (27.6)	
Standardised wealth, mean (SD)	-0.04 (1.0)	0.11 (1.2)	<0.001	0.09 (1.4)	0.07 (1.0)	0.596	0.05 (1.1)	0.12 (1.2)	<0.001	0.04 (1.1)	0.12 (1.2)	<0.001
Diagnosis of:												
Diabetes	286 (9.2)	1270 (11.3)	<0.001	458 (9.7)	1098 (11.5)	0.001	1016 (13.7)	540 (7.8)	<0.001	964 (12.2)	592 (9.2)	<0.001
Cancer	109 (3.5)	683 (6.1)	<0.001	224 (4.7)	568 (5.9)	0.003	504 (6.8)	288 (4.2)	<0.001	410 (5.2)	382 (6.0)	0.044
Lung disease	326 (10.5)	1276 (11.4)	0.192	493 (10.4)	1109 (11.6)	0.036	1106 (14.9)	496 (7.2)	<0.001	879 (11.1)	723 (11.3)	0.743
Cardiovascular condition	462 (14.9)	2346 (20.9)	<0.001	725 (15.3)	2083 (21.8)	<0.001	1936 (26.1)	872 (12.6)	<0.001	1657 (20.9)	1151 (18.0)	<0.001
Psychiatric condition	178 (5.7)	553 (4.9)	0.076	234 (4.9)	497 (5.2)	0.536	458 (6.2)	273 (4.0)	<0.001	393 (5.0)	338 (5.3)	0.417
High cholesterol	669 (21.6)	2613 (23.3)	0.048	1071 (22.6)	2211 (23.1)	0.505	1779 (24.0)	1503 (21.8)	0.002	1843 (23.3)	1439 (22.5)	0.261
High blood pressure	966 (31.2)	4384 (39.1)	<0.001	1684 (35.5)	3666 (38.3)	0.001	3178 (42.9)	2172 (31.5)	<0.001	3064 (38.7)	2286 (35.7)	<0.001

Data shown are N (%) unless otherwise indicated. Pearson's x^2 test used for categorical variables; t-test used for continuous variables. Two-sided p<0.05 considered significant. No correction for multiple comparisons applied.

Abbreviations: SD, standard deviation; MVPA, moderate-vigorous physical activity.

Table S2. Characteristics of the pooled analytic sample at study baseline in women (N=17,716).

	Non-smoking			No-to-moderate alcohol consumption			Weekly MVPA			Weekly social contact		
	No N = 2801	*Yes* N = 14915	P-value	*No* N = 6358	*Yes* N = 11358	P-value	*No* N = 10421	*Yes* N = 7295	P-value	*No* N = 8987	*Yes* N = 8729	P-value
Age at baseline, mean (SD)	59.9 (7.8)	65.1 (10.0)	<0.001	61.4 (8.5)	65.9 (10.3)	<0.001	66.4 (10.4)	61.3 (8.3)	<0.001	64.9 (10.4)	63.6 (9.3)	<0.001
Education												
Below upper secondary	1256 (44.8)	7486 (50.2)		2378 (37.4)	6364 (56.0)		5740 (55.1)	3002 (41.2)		4978 (55.4)	3764 (43.1)	
Upper secondary	1104 (39.4)	5093 (34.1)	<0.001	2514 (39.5)	3683 (32.4)	<0.001	3423 (32.8)	2774 (38.0)	<0.001	2888 (32.1)	3309 (37.9)	<0.001
Tertiary	441 (15.7)	2336 (15.7)		1466 (23.1)	1311 (11.5)		1258 (12.1)	1519 (20.8)		1121 (12.5)	1656 (19.0)	
Standardised wealth, mean (SD)	-0.10 (0.84)	0.02 (1.0)	<0.001	0.05 (1.0)	-0.03 (1.0)	<0.001	-0.04 (0.90)	0.07 (1.1)	<0.001	-0.03 (1.0)	0.04 (1.0)	<0.001
Diagnosis of:												
Diabetes	185 (6.6)	1382 (9.3)	<0.001	359 (5.6)	1208 (10.6)	<0.001	1129 (10.8)	438 (6.0)	<0.001	966 (10.7)	601 (6.9)	<0.001
Cancer	193 (6.9)	1054 (7.1)	0.768	462 (7.3)	785 (6.9)	0.392	829 (8.0)	418 (5.7)	<0.001	589 (6.6)	658 (7.5)	0.011
Lung disease	364 (13.0)	1611 (10.8)	<0.001	694 (10.9)	1281 (11.3)	0.477	1361 (13.1)	614 (8.4)	<0.001	952 (10.6)	1023 (11.7)	0.018
Cardiovascular condition	273 (9.7)	2275 (15.3)	<0.001	649 (10.2)	1899 (16.7)	<0.001	1958 (18.8)	590 (8.1)	<0.001	1348 (15.0)	1200 (13.7)	0.019
Psychiatric condition	350 (12.5)	1537 (10.3)	<0.001	599 (9.4)	1288 (11.3)	<0.001	1232 (11.8)	655 (9.0)	<0.001	1003 (11.2)	884 (10.1)	0.027
High cholesterol	604 (21.6)	3522 (23.6)	0.020	1349 (21.2)	2777 (24.4)	<0.001	2667 (25.6)	1459 (20.0)	<0.001	2161 (24.0)	1965 (22.5)	0.016
High blood pressure	836 (29.8)	6118 (41.0)	<0.001	2111 (33.2)	4843 (42.6)	<0.001	4676 (44.9)	2278 (31.2)	<0.001	3690 (41.1)	3264 (37.4)	<0.001

Data shown are N (%) unless otherwise indicated. Pearson's x^2 test used for categorical variables; t-test used for continuous variables. Two-sided p<0.05 considered significant. No correction for multiple comparisons applied.

Abbreviations: SD, standard deviation; MVPA, moderate-vigorous physical activity.

Table S3. Participants remaining in study at each wave.

	Wave							
	2	3	4	5	6	7	8	9*
**Smoking**								
**Heavy alcohol**								
Less than weekly MVPA								
Less than weekly social contact	748 (100)	487 (65.1)	485 (64.8)	422 (56.4)	376 (50.3)	302 (40.4)	195 (26.1)	14 (1.9)
Weekly social contact	589 (100)	443 (75.2)	409 (69.4)	363 (61.6)	314 (53.3)	259 (44)	200 (34)	96 (16.3)
Weekly MVPA								
Less than weekly social contact	731 (100)	477 (65.3)	477 (65.3)	440 (60.2)	391 (53.5)	326 (44.6)	202 (27.6)	6 (0.8)
Weekly social contact	556 (100)	449 (80.8)	445 (80)	408 (73.4)	355 (63.8)	313 (56.3)	220 (39.6)	56 (10.1)
**No-to-moderate alcohol**								
Less than weekly MVPA								
Less than weekly social contact	1177 (100)	764 (64.9)	756 (64.2)	641 (54.5)	551 (46.8)	446 (37.9)	284 (24.1)	14 (1.2)
Weekly social contact	666 (100)	507 (76.1)	470 (70.6)	422 (63.4)	352 (52.9)	284 (42.6)	212 (31.8)	91 (13.7)
Weekly MVPA								
Less than weekly social contact	876 (100)	596 (68)	593 (67.7)	533 (60.8)	471 (53.8)	392 (44.7)	262 (29.9)	6 (0.7)
Weekly social contact	557 (100)	427 (76.7)	422 (75.8)	377 (67.7)	329 (59.1)	290 (52.1)	204 (36.6)	32 (5.7)
**Non-smoking**								
**Heavy alcohol**								
Less than weekly MVPA								
Less than weekly social contact	1783 (100)	1276 (71.6)	1254 (70.3)	1085 (60.9)	959 (53.8)	807 (45.3)	568 (31.9)	103 (5.8)
Weekly social contact	2403 (100)	2008 (83.6)	1897 (78.9)	1733 (72.1)	1566 (65.2)	1385 (57.6)	1103 (45.9)	671 (27.9)
Weekly MVPA								
Less than weekly social contact	1702 (100)	1231 (72.3)	1222 (71.8)	1116 (65.6)	1004 (59)	879 (51.6)	617 (36.3)	74 (4.3)
Weekly social contact	2589 (100)	2134 (82.4)	2088 (80.6)	1918 (74.1)	1700 (65.7)	1538 (59.4)	1224 (47.3)	461 (17.8)
**No-to-moderate alcohol**								
Less than weekly MVPA								
Less than weekly social contact	6365 (100)	4027 (63.3)	3982 (62.6)	3308 (52)	2866 (45)	2275 (35.7)	1437 (22.6)	95 (1.5)
Weekly social contact	4106 (100)	3233 (78.7)	3018 (73.5)	2677 (65.2)	2337 (56.9)	1974 (48.1)	1491 (36.3)	643 (15.7)
Weekly MVPA								
Less than weekly social contact	3520 (100)	2499 (71)	2494 (70.9)	2202 (62.6)	1921 (54.6)	1651 (46.9)	1096 (31.1)	45 (1.3)
Weekly social contact	3665 (100)	2922 (79.7)	2873 (78.4)	2583 (70.5)	2256 (61.6)	2005 (54.7)	1554 (42.4)	355 (9.7)

*Wave 9 is ELSA only. N (percentage of wave 2) remaining in analytic sample at each wave shown.

Table S4. Mean baseline standardised memory performance in each lifestyle.

	Less than weekly social contact	Weekly social contact
**Smoking**		
Heavy alcohol consumption		
Less than weekly MVPA	0.14 (0.94)	0.13 (0.93)
Weekly MVPA	0.26 (0.90)	0.31 (0.91)
No-to-moderate alcohol consumption		
Less than weekly MVPA	-0.04 (0.94)	0.03 (0.97)
Weekly MVPA	0.13 (0.88)	0.18 (0.90)
**Non-smoking**		
Heavy alcohol consumption		
Less than weekly MVPA	-0.01 (1.00)	0.13 (0.98)
Weekly MVPA	0.17 (0.94)	0.33 (0.94)
No-to-moderate alcohol consumption		
Less than weekly MVPA	-0.33 (1.04)	-0.16 (1.01)
Weekly MVPA	0.02 (0.96)	0.14 (0.95)

Abbreviations: moderate and vigorous physical activity, MVPA.
N (standard deviation) shown.

Table S5. Mean baseline standardised fluency performance in each lifestyle.

	Less than weekly social contact	Weekly social contact
**Smoking**		
Heavy alcohol consumption		
Less than weekly MVPA	0.12 (1.11)	0.04 (1.04)
Weekly MVPA	0.17 (1.01)	0.32 (1.04)
No-to-moderate alcohol consumption		
Less than weekly MVPA	-0.06 (0.91)	-0.05 (0.97)
Weekly MVPA	0.10 (0.90)	0.20 (0.93)
**Non-smoking**		
Heavy alcohol consumption		
Less than weekly MVPA	0.00 (0.97)	0.12 (0.97)
Weekly MVPA	0.17 (1.03)	0.29 (0.99)
No-to-moderate alcohol consumption		
Less than weekly MVPA	-0.32 (0.99)	-0.13 (0.98)
Weekly MVPA	0.01 (0.93)	0.19 (0.98)

Abbreviations: moderate and vigorous physical activity, MVPA.
N (standard deviation) shown.

Table S6. Independent associations between behaviours and cognitive decline.

	**Difference in 10-year cognitive decline (95% confidence interval)**	
	Memory	*Fluency*
Smoking		
Current smoker – not current smoker	0.08 (0.05 to 0.12)	0.08 (0.05 to 0.11)
Current smoker – former smoker	0.09 (0.05 to 0.12)	0.07 (0.03 to 0.11)
Current smoker – never smoker	0.08 (0.04 to 0.12)	0.09 (0.06 to 0.12)
Heavy – no-to-moderate alcohol	0.04 (0.01 to 0.06)	0.03 (0.01 to 0.06)
Less than weekly – weekly MVPA	0.01 (-0.02 to 0.03)	-0.03 (-0.05 to 0.00)
Less than weekly – weekly social contact	0.01 (-0.02 to 0.03)	-0.01 (-0.04 to 0.01)

Based on models adjusted for age at baseline, gender, country, education, wealth, chronic conditions. Positive value indicates recommendation compliant behaviour (non-smoking, no-to-moderate alcohol use, weekly MVPA, weekly social contact) declines less than recommendation non-compliant behaviour.

Table S7. Difference in memory decline over 10 years between the healthiest lifestyle and other lifestyles after excluding participants who abstain from alcohol consumption (N=22,577).

	Less than weekly social contact	Weekly social contact
**Smoking**		
Heavy alcohol consumption		
Less than weekly MVPA	-0.11 (-0.21 to -0.02)	-0.12 (-0.21 to -0.02)
Weekly MVPA	-0.14 (-0.24 to -0.05)	-0.09 (-0.19 to 0.00)
Low-to-moderate alcohol consumption		
Less than weekly MVPA	-0.07 (-0.18 to 0.03)	-0.06 (-0.20 to 0.07)
Weekly MVPA	-0.14 (-0.24 to -0.03)	0.01 (-0.11 to 0.13)
**Non-smoking**		
Heavy alcohol consumption		
Less than weekly MVPA	-0.03 (-0.10 to 0.03)	-0.10 (-0.16 to -0.05)
Weekly MVPA	-0.01 (-0.07 to 0.06)	-0.08 (-0.13 to -0.02)
Low-to-moderate alcohol consumption		
Less than weekly MVPA	0.03 (-0.03 to 0.09)	-0.02 (-0.08 to 0.04)
Weekly MVPA	-0.01 (-0.07 to 0.05)	Reference

Difference in memory decline over 10 years (95% confidence interval) in standard deviations between recommendation compliant lifestyle (reference) and other lifestyles. Negative value indicates memory decline is slower in the reference lifestyle. Based on models adjusted for age at baseline, gender, country, education, wealth, chronic conditions.

Abbreviations: moderate and vigorous physical activity, MVPA.

Table S8. Difference in fluency decline over 10 years between the healthiest lifestyle and other lifestyles after excluding participants who abstain from alcohol consumption (N=22,577).

	Less than weekly social contact	Weekly social contact
**Smoking**		
Heavy alcohol consumption		
Less than weekly MVPA	-0.16 (-0.25 to -0.06)	0.01 (-0.08 to 0.11)
Weekly MVPA	-0.11 (-0.20 to -0.01)	-0.02 (-0.11 to 0.08)
Low-to-moderate alcohol consumption		
Less than weekly MVPA	-0.04 (-0.14 to 0.06)	0.03 (-0.11 to 0.16)
Weekly MVPA	-0.01 (-0.12 to 0.09)	-0.03 (-0.15 to 0.09)
**Non-smoking**		
Heavy alcohol consumption		
Less than weekly MVPA	0.00 (-0.06 to 0.07)	0.01 (-0.04 to 0.07)
Weekly MVPA	0.01 (-0.05 to 0.08)	0.05 (0.00 to 0.11)
Low-to-moderate alcohol consumption		
Less than weekly MVPA	0.08 (0.02 to 0.14)	-0.01 (-0.07 to 0.06)
Weekly MVPA	0.04 (-0.02 to 0.10)	Reference

Difference in fluency decline over 10 years (95% confidence interval) in standard deviations between recommendation compliant lifestyle (reference) and other lifestyles. Negative value indicates fluency decline is slower in the reference lifestyle. Based on models adjusted for age at baseline, gender, country, education, wealth, chronic conditions.
Abbreviations: moderate and vigorous physical activity, MVPA.

Table S9. Difference in memory decline over 10 years between the healthiest lifestyle and other lifestyles after adjustment for practice effect (N=32,033).

	Less than weekly social contact	Weekly social contact
**Smoking**		
Heavy alcohol consumption		
Less than weekly MVPA	-0.15 (-0.24, -0.05)	-0.09 (-0.19, 0.01)
Weekly MVPA	-0.18 (-0.28, -0.09)	-0.08 (-0.18, 0.02)
Low-to-moderate alcohol consumption		
Less than weekly MVPA	-0.11 (-0.19, -0.03)	-0.11 (-0.20, -0.01)
Weekly MVPA	-0.15 (-0.24, -0.06)	-0.00 (-0.11, 0.10)
**Non-smoking**		
Heavy alcohol consumption		
Less than weekly MVPA	-0.05 (-0.11, 0.02)	-0.06 (-0.12, -0.01)
Weekly MVPA	-0.02 (-0.08, 0.05)	-0.03 (-0.09, 0.02)
Low-to-moderate alcohol consumption		
Less than weekly MVPA	-0.02 (-0.07, 0.03)	-0.00 (-0.05, 0.05)
Weekly MVPA	-0.04 (-0.10, 0.01)	

Difference in memory decline over 10 years (95% confidence interval) in standard deviations between recommendation compliant lifestyle (reference) and other lifestyles. Negative value indicates memory decline is slower in the reference lifestyle. Based on models adjusted for age at baseline, practice effect, gender, country, education, wealth, chronic conditions.
Practice effect included as a dichotomous variable showing whether it is a participant’s first round of cognitive testing.
Abbreviations: moderate and vigorous physical activity, MVPA.

Table S10. Difference in fluency decline over 10 years between the healthiest lifestyle and other lifestyles after adjustment for practice effect (N=32,033).

	Less than weekly social contact	Weekly social contact
**Smoking**		
Heavy alcohol consumption		
Less than weekly MVPA	-0.17 (-0.26, -0.08)	0.01 (-0.08, 0.11)
Weekly MVPA	-0.12 (-0.21, -0.03)	-0.02 (-0.11, 0.07)
Low-to-moderate alcohol consumption		
Less than weekly MVPA	-0.05 (-0.13, 0.03)	0.04 (-0.05, 0.13)
Weekly MVPA	-0.04 (-0.12, 0.05)	-0.07 (-0.17, 0.02)
**Non-smoking**		
Heavy alcohol consumption		
Less than weekly MVPA	-0.00 (-0.06, 0.06)	0.02 (-0.04, 0.07)
Weekly MVPA	0.00 (-0.06, 0.07)	0.05 (0.00, 0.10)
Low-to-moderate alcohol consumption		
Less than weekly MVPA	0.10 (0.06, 0.15)	0.04 (-0.01, 0.08)
Weekly MVPA	0.02 (-0.03, 0.07)	Reference

Difference in fluency decline over 10 years (95% confidence interval) in standard deviations between recommendation compliant lifestyle (reference) and other lifestyles. Negative value indicates fluency decline is slower in the reference lifestyle. Based on models adjusted for age at baseline, practice effect, gender, country, education, wealth, chronic conditions.

Practice effect included as a dichotomous variable showing whether it is a participant's first round of cognitive testing.

Abbreviations: moderate and vigorous physical activity, MVPA.

Table S11. Difference in memory decline over 10 years between the healthiest lifestyle and other lifestyles after adjustment for body mass index (N=32,010).

	Less than weekly social contact	Weekly social contact
**Smoking**		
Heavy alcohol consumption		
Less than weekly MVPA	-0.14 (-0.23, -0.04)	-0.09 (-0.19, 0.00)
Weekly MVPA	-0.17 (-0.27, -0.08)	-0.08 (-0.18, 0.02)
Low-to-moderate alcohol consumption		
Less than weekly MVPA	-0.10 (-0.18, -0.02)	-0.11 (-0.21, -0.02)
Weekly MVPA	-0.14 (-0.22, -0.05)	-0.00 (-0.10, 0.10)
**Non-smoking**		
Heavy alcohol consumption		
Less than weekly MVPA	-0.04 (-0.11, 0.02)	-0.07 (-0.13, -0.02)
Weekly MVPA	-0.01 (-0.08, 0.05)	-0.04 (-0.09, 0.02)
Low-to-moderate alcohol consumption		
Less than weekly MVPA	-0.00 (-0.05, 0.04)	-0.01 (-0.06, 0.04)
Weekly MVPA	-0.03 (-0.09, 0.02)	Reference

Difference in memory decline over 10 years (95% confidence interval) in standard deviations between recommendation compliant lifestyle (reference) and other lifestyles. Negative value indicates memory decline is slower in the reference lifestyle. Based on models adjusted for age at baseline, body mass index, gender, country, education, wealth, chronic conditions.
Body mass index recorded at baseline in kg/m^2. Height and weight were measured at a nurse visit in ELSA and were self-reported in SHARE.
Abbreviations: moderate and vigorous physical activity, MVPA.

Table S12. Difference in fluency decline over 10 years between the healthiest lifestyle and other lifestyles after adjustment for body mass index (N=32,010).

	Less than weekly social contact	Weekly social contact
**Smoking**		
Heavy alcohol consumption		
Less than weekly MVPA	-0.16 (-0.25, -0.07)	0.01 (-0.08, 0.10)
Weekly MVPA	-0.11 (-0.20, -0.02)	-0.02 (-0.11, 0.07)
Low-to-moderate alcohol consumption		
Less than weekly MVPA	-0.04 (-0.12, 0.04)	0.04 (-0.05, 0.13)
Weekly MVPA	-0.03 (-0.11, 0.06)	-0.07 (-0.16, 0.02)
**Non-smoking**		
Heavy alcohol consumption		
Less than weekly MVPA	0.00 (-0.06, 0.06)	0.01 (-0.04, 0.06)
Weekly MVPA	0.01 (-0.05, 0.07)	0.05 (0.00, 0.10)
Low-to-moderate alcohol consumption		
Less than weekly MVPA	0.11 (0.07, 0.16)	0.03 (-0.01, 0.08)
Weekly MVPA	0.03 (-0.02, 0.08)	Reference

Difference in fluency decline over 10 years (95% confidence interval) in standard deviations between recommendation compliant lifestyle (reference) and other lifestyles. Negative value indicates fluency decline is slower in the reference lifestyle. Based on models adjusted for age at baseline, body mass index, gender, country, education, wealth, chronic conditions.
Body mass index at baseline in kg/m^2. Height and weight were measured at a nurse visit in ELSA and were self-reported in SHARE.
Abbreviations: moderate and vigorous physical activity, MVPA.

Table S13. Difference in memory decline over 10 years between the healthiest lifestyle and other lifestyles after adjustment for self-reported hearing difficulty (N=32,033).

	Less than weekly social contact	Weekly social contact
**Smoking**		
Heavy alcohol consumption		
Less than weekly MVPA	-0.14 (-0.23, -0.04)	-0.10 (-0.20, 0.00)
Weekly MVPA	-0.17 (-0.26, -0.07)	-0.08 (-0.18, 0.02)
Low-to-moderate alcohol consumption		
Less than weekly MVPA	-0.10 (-0.18, -0.02)	-0.11 (-0.21, -0.02)
Weekly MVPA	-0.14 (-0.22, -0.05)	-0.00 (-0.10, 0.10)
**Non-smoking**		
Heavy alcohol consumption		
Less than weekly MVPA	-0.04 (-0.11, 0.02)	-0.07 (-0.13, -0.02)
Weekly MVPA	-0.01 (-0.07, 0.06)	-0.04 (-0.09, 0.02)
Low-to-moderate alcohol consumption		
Less than weekly MVPA	-0.00 (-0.05, 0.04)	-0.01 (-0.06, 0.04)
Weekly MVPA	-0.03 (-0.09, 0.02)	Reference

Difference in memory decline over 10 years (95% confidence interval) in standard deviations between recommendation compliant lifestyle (reference) and other lifestyles. Negative value indicates memory decline is slower in the reference lifestyle. Based on models adjusted for age at baseline, hearing difficulty, gender, country, education, wealth, chronic conditions.
Participants were asked to report to rate their current hearing (while wearing a hearing aid as applicable) as excellent, very good, good, fair, or poor. Hearing difficulty was included as a continuous variable.
Abbreviations: moderate and vigorous physical activity, MVPA.

Table S14. Difference in fluency decline over 10 years between the healthiest lifestyle and other lifestyles after adjustment for self-reported hearing difficulty (N=32,033).

	Less than weekly social contact	Weekly social contact
**Smoking**		
Heavy alcohol consumption		
Less than weekly MVPA	-0.16 (-0.25, -0.07)	0.01 (-0.08, 0.10)
Weekly MVPA	-0.11 (-0.20, -0.02)	-0.02 (-0.11, 0.07)
Low-to-moderate alcohol consumption		
Less than weekly MVPA	-0.04 (-0.12, 0.04)	0.04 (-0.05, 0.13)
Weekly MVPA	-0.03 (-0.11, 0.06)	-0.07 (-0.16, 0.03)
**Non-smoking**		
Heavy alcohol consumption		
Less than weekly MVPA	0.00 (-0.06, 0.06)	0.01 (-0.04, 0.06)
Weekly MVPA	0.01 (-0.05, 0.07)	0.05 (0.00, 0.10)
Low-to-moderate alcohol consumption		
Less than weekly MVPA	0.11 (0.07, 0.16)	0.03 (-0.01, 0.08)
Weekly MVPA	0.03 (-0.02, 0.08)	Reference

Difference in fluency decline over 10 years (95% confidence interval) in standard deviations between recommendation compliant lifestyle (reference) and other lifestyles. Negative value indicates fluency decline is slower in the reference lifestyle. Based on models adjusted for age at baseline, hearing difficulty, gender, country, education, wealth, chronic conditions.
Participants were asked to report to rate their current hearing (while wearing a hearing aid as applicable) as excellent, very good, good, fair, or poor. Hearing difficulty was included as a continuous variable.
Abbreviations: moderate and vigorous physical activity, MVPA.

Table S15. Countries included in ELSA and SHARE.

Cohort	Region	Country (N, %)
ELSA	Western Europe	England (N=6193, 19.4)
SHARE		Austria (N=917, 2.9)
		Belgium (N=2402, 7.5)
		France (N=2248, 7.0)
		Germany (N=2069, 6.5)
		Netherlands (N=2108, 6.6)
		Switzerland (N=1160, 3.6)
	Northern Europe	Denmark (N=2025, 6.3)
		Sweden (N=2199, 6.9)
	Eastern Europe	Czech Republic (N=2058, 6.4)
		Poland (N=1935, 6.0)
	Southern Europe	Italy (N=2251, 7.0)
		Greece (N=2695, 8.4)
		Spain (N=1773, 5.5)

Abbreviations: ELSA, English Longitudinal Study of Ageing; SHARE, Survey of Health, Ageing and Retirement in Europe
