# Supplementary material for: Healthy lifestyle and cognitive decline in middle-aged and older adults residing in 14 European countries
Source: Nat Commun. 2024 Jun 27;15:5003. doi: 10.1038/s41467-024-49262-5 (PMC11211489; doi:10.1038/s41467-024-49262-5)
Supplement: Supplementary file 2 — Reporting Summary [file 41467_2024_49262_MOESM2_ESM.pdf]

nature portfolio

Corresponding author(s): Mikaela Bloomberg

Last updated by author(s): Apr 8, 2024

Reporting Summary

Nature Portfolio wishes to improve the reproducibility of the work that we publish. This form provides structure for consistency and transparency in reporting. For further information on Nature Portfolio policies, see our Editorial Policies and the Editorial Policy Checklist.

Statistics

For all statistical analyses, confirm that the following items are present in the figure legend, table legend, main text, or Methods section.

n/a	Confirmed	
☐	☒	The exact sample size (*n*) for each experimental group/condition, given as a discrete number and unit of measurement
☐	☒	A statement on whether measurements were taken from distinct samples or whether the same sample was measured repeatedly
☐	☒	The statistical test(s) used AND whether they are one- or two-sided<br>*Only common tests should be described solely by name; describe more complex techniques in the Methods section.*
☐	☒	A description of all covariates tested
☐	☒	A description of any assumptions or corrections, such as tests of normality and adjustment for multiple comparisons
☐	☒	A full description of the statistical parameters including central tendency (e.g. means) or other basic estimates (e.g. regression coefficient) AND variation (e.g. standard deviation) or associated estimates of uncertainty (e.g. confidence intervals)
☐	☒	For null hypothesis testing, the test statistic (e.g. *F*, *t*, *r*) with confidence intervals, effect sizes, degrees of freedom and *P* value noted<br>*Give P values as exact values whenever suitable.*
☒	☐	For Bayesian analysis, information on the choice of priors and Markov chain Monte Carlo settings
☒	☐	For hierarchical and complex designs, identification of the appropriate level for tests and full reporting of outcomes
☐	☒	Estimates of effect sizes (e.g. Cohen's *d*, Pearson's *r*), indicating how they were calculated

Our web collection on statistics for biologists contains articles on many of the points above.

Software and code

Policy information about availability of computer code

Data collection	Blaise
Data analysis	StataMP version 18

For manuscripts utilizing custom algorithms or software that are central to the research but not yet described in published literature, software must be made available to editors and reviewers. We strongly encourage code deposition in a community repository (e.g. GitHub). See the Nature Portfolio guidelines for submitting code & software for further information.

Data

Policy information about availability of data

All manuscripts must include a data availability statement. This statement should provide the following information, where applicable:

- Accession codes, unique identifiers, or web links for publicly available datasets
- A description of any restrictions on data availability
- For clinical datasets or third party data, please ensure that the statement adheres to our policy

ELSA data are available to researchers after registration with the UK data service; see: https://www.elsa-project.ac.uk/accessing-elsa-data. SHARE data are accessible after registration with the SHARE project at the following addresses at https://share-eric.eu/data/data-access.

Research involving human participants, their data, or biological material

Policy information about studies with human participants or human data. See also policy information about sex, gender (identity/presentation), and sexual orientation and race, ethnicity and racism.

Reporting on sex and gender	Gender was self-reported as man or woman in ELSA, with 14,317 men (44.7%) and 17,716 women (53.1%) included in the analytic sample. We examined interaction terms between lifestyle and gender and determined that results for associations between lifestyle and cognitive decline were similar for men and women; as such analyses were performed without stratifying by gender.
Reporting on race, ethnicity, or other socially relevant groupings	Covariates were selected on the basis of previous evidence of associations with lifestyle and cognitive function and were ascertained by self-report. Sociodemographic covariates included gender (man or woman), age at baseline in years, and country. Socioeconomic covariates included education (less than upper secondary, upper secondary, or tertiary and above; categorised based on the International Standard Classification of Education 2011) and household non-housing wealth.
Population characteristics	32,033 cognitively-healthy adults aged 50-104 years from 14 European countries.
Recruitment	Data were drawn from the English Longitudinal Study of Ageing (ELSA) and the Survey of Health, Aging and Retirement in Europe (SHARE). ELSA is a nationally representative cohort study of community-dwelling adults aged 50+ years residing in England. The ELSA sampling frame was drawn from the Health Survey for England years 1998, 1999, and 2001. SHARE is a nationally representative cohort study of community-dwelling adults aged 50+ years residing in 28 European countries and Israel. The SHARE sampling frame is developed using national registers available in each country (e.g, telephone registers).
Ethics oversight	ELSA Wave 9 received ethical approval from the South Central – Berkshire Research Ethics Committee on 10th May 2018 (17/SC/0588). ELSA Wave 8 received ethical approval from the South Central – Berkshire Research Ethics Committee on 23rd September 2015 (15/SC/0526). ELSA Wave 7 received ethical approval from the NRES Committee South Central - Berkshire on 28th November 2013 (13/SC/0532). ELSA Wave 6 received ethical approval from the NRES Committee South Central - Berkshire on 28th November 2012 (11/SC/0374). ELSA Wave 5 received ethical approval from the Berkshire Research Ethics Committee on 21st December 2009 (09/H0505/124). ELSA Wave 4 received ethical approval from the National Hospital for Neurology and Neurosurgery & Institute of Neurology Joint Research Ethics Committee on 12th October 2007 (07/H0716/48). ELSA Wave 3 received ethical approval from the London Multi-Centre Research Ethics Committee on 27th October 2005 (05/MRE02/63). ELSA Wave 2 received ethical approval from the London Multi-Centre Research Ethics Committee on 12th August 2004 (MREC/04/2/006). Ethical approval of SHARE from first to fourth waves was obtained from the Ethics Committee of the University of Mannheim. Most recently, in 2021, the Ethics Council of the Max Planck Society reviewed and approved the fourth and the consecutive waves of the SHARE project. No further ethical approval was required for the current study.

Note that full information on the approval of the study protocol must also be provided in the manuscript.

Field-specific reporting

Please select the one below that is the best fit for your research. If you are not sure, read the appropriate sections before making your selection.

☐ Life sciences ☒ Behavioural & social sciences ☐ Ecological, evolutionary & environmental sciences

For a reference copy of the document with all sections, see nature.com/documents/nr-reporting-summary-flat.pdf

Behavioural & social sciences study design

All studies must disclose on these points even when the disclosure is negative.

Study description	Quantitative
Research sample	32,033 cognitively healthy adults aged 50-104 years from 14 European countries participating in at least wave 2 of ELSA (2004/05) or SHARE (2006/07). The analytic sample for the present study is a non-representative sub-sample of nationally-representative cohort studies. SHARE and ELSA were chosen to maximise the number of included European countries whilst maintaining harmonisation of cognitive measures. Participants showing evidence of cognitive impairment or who reported dementia diagnosis were excluded to reduce the impact of cognitive dysfunction on lifestyle behaviours.
Sampling strategy	The present study is a secondary analysis of two ongoing nationally-representative cohort studies of ageing (ELSA and SHARE). ELSA and SHARE used multistage stratified random sampling to identify households for participation. Eligibility criteria for ELSA were as follows: membership of a participating household from HSE in which at least one person had agreed to follow-up, aged 50 years and above, and living in a private household in England at the time of the first wave of fieldwork. The SHARE target population consists of all persons aged 50 years and above who have their regular domicile in the respective SHARE country. A person is excluded if she or he is incarcerated, hospitalised or out of the country during the entire survey period, unable to speak the country's language(s) or has moved to an unknown address. Initial sample sizes were selected to give an adequate number of men and women in 5-year age bands; pooling of ELSA and SHARE data gave sufficient sample size to examine lifestyle differences in cognitive decline.
Data collection	ELSA and SHARE interviews take place in person with a trained interviewer using a combination of self-completed questionnaires and computer-assisted personal interviewing.

Timing	The present analysis included waves 2-8 of ELSA (January 2004-July 2019), and waves 2 and 4-8 of SHARE (January 2006-March 2020), comprising up to 15 years of follow-up. Wave 3 of SHARE is a life history module that is not part of the main survey.
Data exclusions	Exclusion criteria were pre-established. Of 9,131 respondents aged 50 years and above participating in wave 2 of ELSA, 1,088 (11.9%) were missing more than one behaviour at baseline and therefore did not have missing behaviour data imputed, 1,821 (19.9%) either reported dementia diagnosis or had cognitive scores suggesting cognitive impairment, 5 (<0.1%) were missing cognitive scores at all waves of follow-up, and 24 (0.3%) were missing covariates; these respondents were excluded leading to 6,193 ELSA respondents being included in analyses. Of 32,657 respondents aged 50 years and above participating in wave 2 of SHARE, 125 (0.4%) had alcohol top-coded at 70 drinks per day, 595 (1.8%) were missing more than one behaviour, 5,962 (18.3%) reported dementia diagnosis or had cognitive scores suggesting cognitive impairment, 109 (0.3%) were missing cognitive scores at all waves, and 26 (<0.1%) were missing covariates; these respondents were excluded leading to 25,840 SHARE respondents being included in analyses.
Non-participation	Of 32,033 participants in wave 2 (the baseline wave), 23480 (73.3%) were in the study at wave 3, 22885 (71.4%) at wave 4, 20228 (63.1%) at wave 5, 17748 (55.4%) at wave 6, 15126 (47.2%) at wave 7, and 10869 (33.9%) at wave 8. Missing individuals were either deceased, lost to follow up, or missing lifestyle, cognitive function, or covariate data for all subsequent waves.
Randomization	Randomisation was not possible due to the observational nature of the study. Covariates were selected on the basis of previous evidence of associations with lifestyle and cognitive function and were ascertained by self-report. Sociodemographic covariates included gender (man or woman), age at baseline in years, and country. Socioeconomic covariates included education (less than upper secondary, upper secondary, or tertiary and above; categorised based on the International Standard Classification of Education 2011) and household non-housing wealth. Wealth was standardised to each country by year and converted into quintiles, with the highest quintile corresponding to the greatest wealth. Chronic conditions included high blood pressure, diabetes, cardiovascular conditions (including heart disease and stroke), cancer, lung disease, high cholesterol, and psychiatric conditions. These covariates were included in the analytical models to account for confounding.

Reporting for specific materials, systems and methods

We require information from authors about some types of materials, experimental systems and methods used in many studies. Here, indicate whether each material, system or method listed is relevant to your study. If you are not sure if a list item applies to your research, read the appropriate section before selecting a response.

Materials & experimental systems

n/a	Involved in the study	
☒	☐	Antibodies
☒	☐	Eukaryotic cell lines
☒	☐	Palaeontology and archaeology
☒	☐	Animals and other organisms
☒	☐	Clinical data
☒	☐	Dual use research of concern
☒	☐	Plants

Methods

n/a	Involved in the study	
☒	☐	ChIP-seq
☒	☐	Flow cytometry
☒	☐	MRI-based neuroimaging

Plants

Seed stocks	N/A
Novel plant genotypes	N/A
Authentication	N/A
